# The Efficacy of Ultramolecular Aqueous Dilutions on a Wheat Germination Model as a Function of Heat and Aging-Time

**DOI:** 10.1093/ecam/nep217

**Published:** 2011-02-14

**Authors:** Maurizio Brizzi, Vittorio Elia, Grazia Trebbi, Daniele Nani, Maurizio Peruzzi, Lucietta Betti

**Affiliations:** ^1^Department of Statistical Sciences, University of Bologna, Bologna, Italy; ^2^Department of Chemistry, “Federico II” University of Naples, Naples, Italy; ^3^Department of Agri-Environmental Sciences and Technologies, University of Bologna, Bologna, Italy; ^4^Italian Society of Anthroposophical Medicine, Milan, Italy; ^5^Association for Sensitive Crystallization, Milan, Italy; ^6^Lucietta Betti, Dipartimento di Scienze e Tecnologie Agroambientali, Viale Fanin 42, 40127 Bologna, Italy

## Abstract

This study evaluates the effects of temperature and aging on the efficacy of As_2_O_3_ at the 45th decimal potency in a wheat germination model, compared against a control and potentized H_2_O 45×. Each treatment-temperature combination was tested on seeds (*Triticum aestivum* L.) of Pandas variety, using six Petri dishes (33 seeds/dish) per trial, performing eight trials. Seeds were pre-treated by poisoning with 0.1% As_2_O_3_ solution to reduce germination, to allow a better evaluation of homeopathic treatment effects. The outcome variable was the number of non-germinated seeds after 96 h. Temperature effect was investigated by heating each treatment in a water bath for 30 min (at 20, 40 or 70°C), or for 5 min (at 100°C), and that of aging by dividing experimental data, collected over a period of nearly five months, into two groups: early and late experiments. Results seem to show that the efficacy of As_2_O_3_ 45× is unaltered at 20 and 40°C, increases at 70°C and decreases at 100°C. As regards aging, a notable difference was found between early trials, with no significant efficacy, and late trials, where As_2_O_3_ 45× exhibits a repeated significant effect versus control, except at 100°C. A reduction in variability was observed for As_2_O_3_ 45× at 20°C versus control, confirming the findings of previous work. The main conclusion suggested by this experiment is that the efficacy of As_2_O_3_ 45× on wheat germination may be influenced by heating degree and seems to have an increasing trend as a function of aging.

## 1. Introduction

Homeopathy has always been a subject of controversy, and the debate has been recently taken up by a meta-analysis published in *The Lancet* [1–5]. The discussion about this form of complementary medicine centers essentially on the specific effects of ultramolecular dilutions (beyond the Avogadro limit), which are judged to be “implausible” according to conventional science, although there is emerging evidence for *in vitro* activity of ultra-high dilutions [6–11]. For homeopathy to become accepted as a valid part of medical practice, its scientific bases must be rigorously assessed by various experimental approaches.

To this end, plant- and micro-organism-based experiments appear especially well suited, since they allow some of the drawbacks of clinical trials to be overcome. Botanical and microbial trials are not susceptible to the placebo effect nor to ethical problems, and rely on a very cheap and almost inexhaustible source of biological material [12, 13]. What is more, they allow relatively simple model systems to be adopted, so that a more direct treatment/effect relationship and large data samples for structured statistical analysis can be obtained. By reducing complexity, plant-based bioassays permit a higher degree of standardization than does clinical research, and their ability to yield large numbers of experimental repetitions and external replications can help address the problem of non-reproducibility that so often arises in the homeopathic literature [14, 15]. Furthermore, since the main cell structures and functions are common to the majority of eukaryotes [16, 17], plant and eukaryotic microbial bio-assays have potentially relevant implications for therapeutic applications.

A well-known homeopathic remedy, *Arsenicum album*, prepared starting from arsenic trioxide (As_2_O_3_), was used by our research group in a series of experiments on an isopathic model, based on *in vitro* wheat germination. According to `isopathy', the same substance causing the disease can be used in low doses or high dilutions to treat the disease itself [18]; this concept is analogous to the hormesis effect [8]. In our model, a large number of wheat seeds were stressed with a sub-lethal dose of As_2_O_3_ and then treated with decimal potencies of the same substance [19–22]. The consistency of the different statistical analyses and the reproducibility of most of the experimental results are notable. In particular, the As_2_O_3_ 45× potency always induced a significant stimulating effect, compared to both the control and to H_2_O of the same potency, whereas As_2_O_3_ diluted to 10^−45^ without potentization never showed any significant effect. These results seem to indicate that the potentization process is essential for obtaining an enhanced biological efficacy relative to the control. It is worth to point out that 45× potency is an ultramolecular dilution, well beyond the Avogadro's limit and that arsenic molecules from mother tincture are no more present; however, since arsenic is ubiquitous in the environment, a very low level of contamination may be present.

The topic of *in vitro* wheat seedling growth has also been jointly investigated by two independent research groups: the results of the replication trial conducted by Binder [23] are the reverse of those of the original study [20], with As_2_O_3_ 45× inhibiting wheat shoot growth instead of enhancing it, while the replication trail conducted by Betti's research group [22] confirmed the result of the original study [20]. Despite these discrepancies, high homeopathic potencies did induce statistically significant effects in both experiments, even if the magnitude and direction of such effects seem to depend on as yet unknown parameters [23]. An elucidation of the factors responsible for the size and direction of the effects would yield important insights, possibly helping to account for the reproducibility problems also encountered in other systems.

The primary aim of the study described here, adopting the aforesaid isopathic model, is to investigate whether “treatment heating” or “treatment aging time” can affect the efficacy of As_2_O_3_ 45× on *in vitro* wheat germination. In this connection, we recall that a Naples University research group, in a systematic study of the physical-chemical properties of ultra-high aqueous dilutions as a function of the time parameter, has already shown that the process of iterated dilution and succussion is able to permanently modify certain features of water [24–27]. A second aim of the present work is to confirm our previous finding that one of the peculiar effects of ultramolecular dilutions is to systematically reduce variability [22, 28, 29].

## 2. Methods

### 2.1. Biological Model and Classes of Treatment

The experiment was performed at the Urania Laboratory, located in Milan (Italy), and consisted of 16 trials, carried out weekly from November to April, using wheat seeds (*Triticum aestivum* L.) of the Pandas variety. The seeds were pre-treated by 30 min of poisoning with 0.10% arsenic trioxide aqueous solution (AS_2_O_3_, Aldrich, St. Louis, MO, USA), and then rinsed in tap water for 60 min, dried in ambient air until they reached 12% moisture content and stored in the dark at room temperature until use. This stress was defined after performing a series of trials, as already reported [20], in which we tested arsenic dilutions (from 0.02% to 0.20%) for different exposure times (from 30 to 120 min), and resulted to be the highest sublethal dose. Non-stressed controls showed a mean germination rate of 94.6% [19, 21]; the selected stress reduced the germination rate approximately by 15% with respect to non-stressed controls, obtaining a germination rate of ~80% [21]. Actually, with an arsenic concentration of 0.15% (instead of 0.10%), applied for the same time, germination rate fell down to <10%.

Three classes of treatments were then applied to the stressed seeds:

- Pure water (p.A., Merck, Darmstadt, Germany) (C, Control),
- Pure water at the 45th decimal potency (H_2_O 45×) and
- As_2_O_3_, at the 45th decimal potency (As 45×).

As 45× treatment (containing a theoretical dose of As_2_O_3_ 1 × 10^−47^ M) was obtained as described in a previous contribution [22], through serial dilution (1 : 10) with pure water (p.A., Merck) and succussion, starting from a 0.20% solution of As_2_O_3_ (0.01 M). The dynamization was performed using a specially designed succussion machine that vertically shakes 1000 ml volumes (in polyethylene bottles filled to 90% of capacity) at a rate of 70 times per min with an oscillation amplitude of 24 cm; each potency was succussed for 1 min. Potentized water was prepared using exactly the same method of serial dilution and succussion, with the only difference that there was no arsenic in the starting solution. For each class of treatments, 4 l have been prepared and soon afterward divided in four equal amounts (1 l per glass bottle), which were then heated in a water bath, respectively, at 20, 40, 70 (for 30 min) and at 100°C (for 5 min). The different treatments were then poured in polyethylene bottles, letter-coded according to a blind protocol, by a person not involved in the experiments. In order to reduce microbial growth, bottles were stored at a cool temperature (4°C) until use.

We focused our attention on seed germination *in vitro*, applying the same biological pattern adopted in previous studies [19, 21]: a fixed number of 33 wheat seeds were selected for integrity and placed, median groove upward, in two concentric circles on sterilized sand in 10-cm-diameter Petri dishes. At the beginning of each experiment, a fixed quantity of treatment (20 ml) was pipetted into each dish, without disturbing the seeds. Petri dishes were then labeled (coding treatments) and randomly distributed, following a circular pattern, in a germination box with a wooden base and glass walls and cover that was mounted on an electrically driven plate rotating at 90 rpm. Dishes were kept at room temperature (20°C), in daylight and at a constantly high humidity rate (~70% relative humidity). This procedure was followed to obtain maximum homogeneity of experimental conditions. At the end of each experiment, ungerminated seeds were counted by an experimenter which was blind to group allocation.

In order to evaluate the stability of experimental set-up, we performed a preliminary set of five independent and systematic negative control experiments using pure, unsuccussed water (p.A., Merck) as the only test substance. In each experiment, 6 Petri dishes with 33 stressed seeds per each temperature (20, 40, 70 and 100°C) were considered, following the same experimental protocol and evaluating the same outcome variable as above described.

### 2.2. Experimental Variables

**Figure 1:**
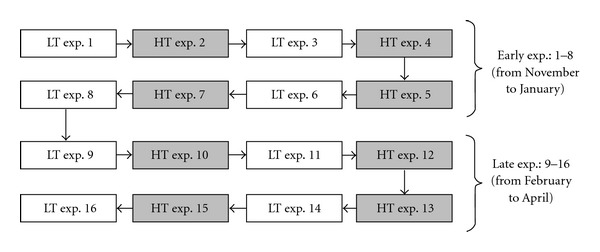
Flowchart of all 16 experiments analysed in the present study, one box corresponding to a single experiment. LT = low temperatures (C, H_2_O 45×, As 45× at 20° and 40°C); HT = high temperatures (C, H_2_O 45×, As 45× at 70° and 100°C); exp = experiment;→ = time-sequence of the experiments.

In this work, we investigated the independent variables of temperature (treatment heating) and time (treatment aging) to ascertain how they influence treatment efficacy on wheat seed germination. This was accomplished by testing the three treatment classes (C, H_2_O 45×, As 45×) in combination with four different temperatures (20, 40, 70, 100°C). Trials were conducted on sets of 36 Petri dishes, equally divided among the three treatments (12 C, 12 H_2_O 45×, 12 As 45×), each dish yielding a germination datum. In each trial, two distinct temperatures were investigated, so that there were six dishes per treatment at each temperature. As shown in Figure 1, a total of 16 experiments were performed, alternating evenly between trials at the two “low” temperatures (20 and 40°C) and trials at the two “high” temperatures (70 and 100°C), thus allowing each treatment and temperature combination to be observed in eight different trials, yielding a total of 48 results for each combination. The treatment aging effect was tested by dividing the samples from the 16 trials into two groups, separating early experiments (first eight trials) from late experiments (last eight trials).

### 2.3. Statistical Analysis

As already mentioned, the outcome variable of the experiments was the number, denoted with *X*, of non-germinated seeds (out of 33) after 96 h of observation. We decided, as done in previous works [19, 21] to use this outcome variable because it fits very well in a Poisson distribution, either in treatment or in control groups. Then, combining the four different temperatures (20, 40, 70, 100°C) with the three classes of treatment (C, H_2_O 45×, As 45×), we obtain 12 distinct experimental conditions; for each of which a sample of 48 Petri dishes was collected, having a total of 576 data points.

First of all, we applied a global Poisson test for negative control experiments. Such test allows us to compare simultaneously *k *(>2) Poisson parameters, and its test statistic follows, under null hypothesis of perfect equality of parameters, a *χ* ^2^ distribution with *k* − 1 degrees of freedom, where *k* is the number of parameters compared [30]. After this check, we calculated, for each sample, the usual exploratory statistics such as average, median, standard deviation (SD) and mean absolute deviation from the median (MAD). We detected a good fit of the data to the Poisson distribution, which confirms the results reported in previous works [19, 21]. We applied again a global Poisson test, as previously done with control data, comparing all the 12 experimental conditions at the same time. The same test was then performed on pairs of treatment classes (C + H_2_O 45×, C + As 45×, H_2_O 45× + As 45×), at all the temperatures. After checking the global significance, a pairwise Poisson test was applied to compare the control (C) and treatment (H_2_O 45×, As 45×) groups at the same temperature. The pairwise comparisons were also checked by the Wilcoxon-Mann-Whitney rank sum test for independent samples. This non-parametric test was applied taking into account the markedly non-normal distribution of the data. A pairwise Poisson test was also employed to separately analyze the early trials (from 1st to 8th) and late trials (from 9th to 16th), in order to evaluate the treatment aging effect. The aging effect was then investigated more in detail, only for the 20°C temperature, by computing cumulative averages of the variable *X*, starting by considering the first trial only, then the first and second trial, and so forth, up to the full set of eight trials per treatment at that temperature. The cumulative trend for *X* was then summarized by linear interpolation, and the Bravais-Pearson linear coefficient of correlation *r* = (Cov ((*X*, *Y*)/SD(*X*)SD(*Y*)) computed to determine the degree of linearity.

Finally, the effect of the investigated treatments on variability was evaluated in terms of the SD, divided into two components (SD within and between experiments). This analysis was restricted to the 20°C group, in order to permit comparisons with the results of previous work [29].

## 3. Results

### 3.1. Stress Evaluation

A stress with As_2_O_3_ at 0.10% has been chosen, as above reported, since a stronger concentration induced a decrease in germination rate too strong to allow a possible recovery due to consequent homeopathic treatment.

Having the aim of pointing out the heuristic properties of an isopathic model, we report (Table 1) a brief summary of our previous data [19, 21], representing the comparison between the effects of As 45× on the germination of non-stressed and stressed seeds. We can observe that relative effect of homeopathic treatment sensibly increases when working with stressed seeds.

### 3.2. Negative Control Experiments

We began our statistical evaluation investigating whether there were any differences between control groups at different temperatures within the same experiment (six Petri dishes per each temperature = 198 total seeds), as well as between experiments at the same temperature. The results of the global Poisson test are reported in Table 2 and did not evidence any significance whatsoever, thereby confirming that the experimental set-up was stable and did not generate false positive results.

### 3.3. Exploratory Statistics and Global Comparisons

**Figure 2:**
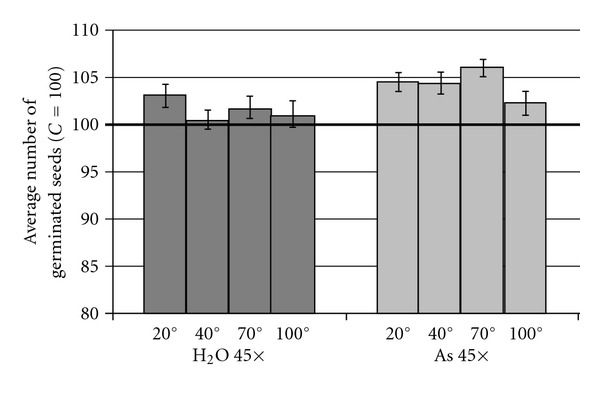
Average number of germinated seeds at each temperature (control = 100). Bars indicate standard errors of average values.

After this preliminary check on controls, we computed a set of exploratory statistics (Table 3) that provided an initial visual impression of how the studied treatments affected wheat germination. It can be clearly seen that the average number of non-germinated seeds is higher in control groups, at all temperatures, whereas it is lowest in all groups treated with As 45× (i.e., a higher germination versus control). For every class of treatment, SD appears to be influenced by temperature. Another notable feature is that at 20°C (standard ambient temperature), As 45× shows a marked decrease in variability when compared to control. A similar trend emerges looking at the median and MAD. In particular, to simplify the visual impression and interpretation of our results, in Figure 2 we represented the average values of germinated seeds normalized against the corresponding control values set equal to 100; here the As 45× treatment class has the most noticeable effects, with a germination increase of ~5% at 20, 40 and 70°C, while the only relevant effect observed for the H_2_O 45× class is at 20°C with a germination increase of ~3%.

Before doing the pairwise comparisons, a global Poisson test (Table 4) was performed to simultaneously compare all the 12 treatment/temperature combinations (C + H_2_O 45× + As 45× at 20, 40, 70 and 100°C). Since the outcome of this test was significant (*P *< .01), we applied the same test again, but to all possible pairs of treatment groups at every temperature (C + H_2_O 45×, C + As 45×, H_2_O 45× + As 45× at 20, 40, 70 and 100°C). The highest significance (*P *< .001) was found for the C + As 45× multiple comparison, though it is worth noting that the H_2_O 45× + As 45× comparison was also significant (*P *< .05). These results are evidence for the possible existence of a global effect associated with the combined treatments, each determined by class of treatment and temperature.

### 3.4. Efficacy of Potentized Treatment Classes as a Function of Heat

After the multiple statistical comparison of treatments, pairwise comparisons between treatments were performed using the Poisson test for two samples.  Table 5 shows the significance results for all the 12 possible pairs. The most notable of these is the repeatedly high significance versus control of the As 45× treatment class at 20, 40 and 70°C (*P *< .01), which appears to lose its effectiveness only after a severe stress (100°C). The H_2_O 45× treatment is instead significant versus control only at 20°C (*P *< .05), and loses its efficacy at higher temperatures; as a consequence of this, the comparison between H_2_O 45× and As 45× is highly significant (*P *< .01) only at 40 and 70°C.

The above reported results are confirmed by the rank sum test (Table 5) with only one exception, for the H_2_O 45× versus C comparison at 20°C, which proves not to be significant. However, taking into account that the Wilcoxon-Mann-Whitney test is slightly less powerful than a parametric test, the *P*-value of around 0.07 yielded by this comparison is not so far from significance threshold. Finally, we note that the strongest significance level occurs at 70°C when testing As 45× versus control, especially when using the rank sum test (*P *< .0001). This peculiar result may be partially explained with the slightly lower germination rate of controls at 70°C, as noticeable by the higher number of non-germinated seeds in Table 3. We also compared each class of treatment, at different temperatures, between themselves, both with Poisson and rank sum test, but no significance was found at all.

### 3.5. Efficacy of Potentized Treatment Classes As a Function of Aging-Time

Next, given that the study was carried out over a period of nearly 5 months, we also attempted to analyze the effects of treatment aging on its efficacy. To this end, the set of experimental data was divided into two groups, early trials (1–8, from November to January) and late trials (9–16, from February to April), applying the Poisson test, as reported in Table 6. We can see that there is a marked difference between early trials, whose results are never significant, and late trials, in which As 445× treatments do show a repeated and strong significance versus control (*P *< .001 at 20 and 40°C, *P *< .01 at 70°C). Moreover, the comparison of As 45× versus H_2_O 45× is significant at 40°C (*P *< .01) and 70°C (*P *< .05).

**Figure 3:**
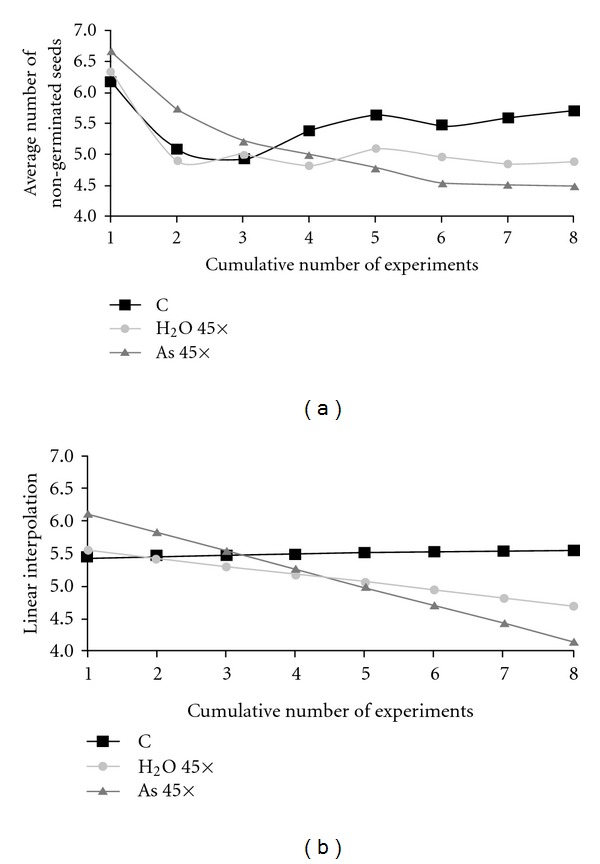
Cumulative trend for the average number of non-germinated seeds at 20° C (from November to April); (a): observed cumulative average values; (b): linear interpolation of cumulative average values.

In order to study treatment aging effect more in detail, we focused our attention essentially on the subset of data at 20°C, the standard ambient temperature, and computed for this data set the cumulative averages of non-germinated seeds, considering now all the eight experiments carried out at 20°C. These averages are graphically plotted in Figure 3(a) (observed data) and 3(b) (linear interpolation). These reveal clear differences between the studied treatments: the control has an oscillatory trend, evinced by a nearly constant linear interpolation (*r*  =  +0.091), H_2_O 45× has a moderately but clearly decreasing trend (*r*  =  −0.593), whereas As 45× follows a regular and almost linear decreasing trend (*r*  =  −0.909).

### 3.6. Variability Analysis

Finally, we investigated variability at 20°C, splitted into its two components (within and between experiments), expressed in terms of SD (Table 7). Considering all the trials together, both As 45× and H_2_O 45× treatments show a clear reduction in both variability components compared to the control. The effect is stronger for the As 45× treatment, for which the overall decrease in SD versus control is close to 25%, while the corresponding decrease for the H_2_O O 45× treatment is just above 6%. We also attempted to investigate the effect of aging on variability, by comparing early and late trials. However, it should be pointed out that the sample size for this analysis is very small, especially when looking at the variability between trials (only four data points). Bearing this in mind, we can nevertheless make a few considerations: the As 45×> treatment appears to be associated with a marked decrease in variability for both early trials and late trials, whereas the H_2_O 45× treatments show some effect on variability only in early trials. In particular, the SD between trials is almost halved (−49.1%) for the As 45× treatment in late trials.

## 4. Discussion

The isopathic approach is generally used to test homeopathic specific effects in low potencies that can influence plants due to molecular-non-homeopathic effects; given a pre-existing damage by higher concentrations of the same substance, any effect of a treatment with lower concentrations cannot be explained by the material presence of this substance [13]. In the present study we used an ultramolecular dilution (45× potency) of *Arsenicum album*, well beyond the Avogadro's limit. Nevertheless, we adopted an isopathic model because in previous works we observed an “isopathic sensitization,” that is, a sensible increase of homeopathic treatment effects when working with stressed seeds (Table 1; [19, 21].

As far as the effects of ponderal arsenic on wheat plants are concerned, a large number of studies indicate that low concentrations stimulate seed germination and root/shoot growth; however, these factors all decrease at a high concentration of arsenic [31, 32]. Moreover, some physiological activities such as the contents of superoxide anion-free radical, malondialdehyde, acetylsalicylic acid, soluble sugar and protein, and chlorophyll, as well as some enzymatic activities (ascorbate peroxidase, superoxide dismutase and catalase), were differently affected by arsenic in leaves of wheat seedlings [32].

**Figure 4:**
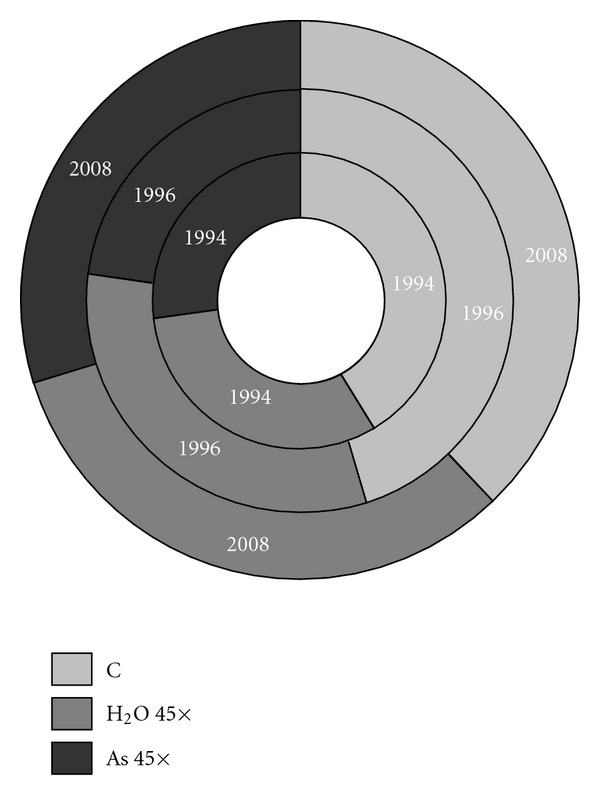
Ring diagram for the comparison between present data (2008) and previously published ones (1994 and 1996): the average number of non-germinated seeds in the control, H_2_O 45× and As 45× treatment groups at 20°C is shown.

Referring now to our findings, the first general consideration we can make is that the results for the 20°C temperature are strongly consistent with those previously published [21]: the average number of non-germinated seeds after 4 days of observation is very similar, particularly in the control group, but also satisfactorily so in the 45× potentized arsenic treatment group (Figure 4). The data for SD are likewise consistent with previously published results [29]. This reproducibility seems to suggest a specific effect of As 45× potency and confirms that the *in vitro* wheat germination model may be suitable for further studies on the efficacy of ultramolecular dilutions. Plant-based research in homeopathy has also shown that other models can be successfully adopted for the study of high dilution effects: in particular, some potency levels of gibberellic acid have been found to induce reproducible effects on dwarf pea shoot growth [33] and on the growth rate of *Lemna gibba* [34], with a different directions of the effect (increasing or decreasing, resp.), depending on the state of the system.

The present study has found a marked stimulating effect, in terms of the reduction in the number of non-germinated seeds, for As 45× at 20°C (−21.2%): this percentage is consistent with the values reported in our previous papers [20–22] and in other studies on intoxication models [35, 36], whereas in healthy test plants the effects of potentized substances are less pronounced [21, 34, 37–39].

As far as potentized water at the 45th decimal is concerned, the significance of its effect versus control at 20°C, determined by application of the Poisson test, seems in line with our previous findings [21], with a *P*-value of.042 (Figure 4). However, it should be noted that with the Wilcoxon–Mann–Whitney rank sum test (less powerful, like all non-parametric tests), the *P*-value instead exceeds the classic significance limit of.05 (*P* = .072), a result consistent with the findings of other authors [33, 34], who report no significant effects of potentized water on any plant growth parameter. On the other hand, the electrochemical behavior of H_2_O 45× strongly supports, at least from a physical-chemical point of view, the significance indicated by the Poisson test [40].

**Figure 5:**
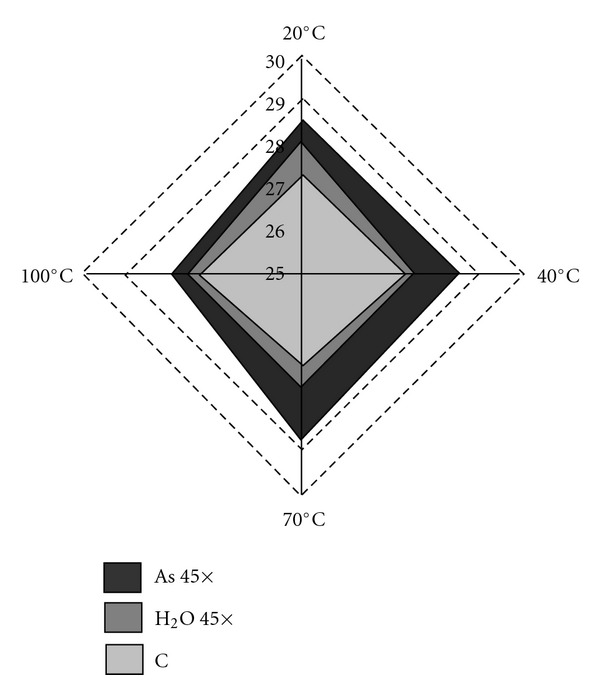
Radar diagram showing the temperature effect (20°, 40°, 70° and 100°C) on the average number of germinated seeds in control, H_2_O 45× and As 45× treatment groups.

Turning now to consider the temperature effect, we notice that the efficacy of As 45× is not altered by heating to 40°C and slightly increases by heating to 70°C, sensibly reducing its efficacy at 100°C. On the other hand, H_2_O 45× has no effect at any temperature above 20°C (Figure 5). We can hypothesize that the absence of active principle at the start of the potentization process for obtaining potentized water induces modifications in the water structure that are unstable, at least as the temperature increases [25]. Contrariwise, the efficacy of As 445× even at 40 and 70°C (with a slight rise in efficacy at 70°C) may be accounted for by a heat-induced increase in kinetic variation of the supramolecular structure of water molecules in the As 45× treatment. This hypothesis seems to be borne out by preliminary physical-chemical studies on the effect of temperature increases (from 25 to 37°C) on electrical conductivity (Elia 2009, personal communication). The decrease of efficacy at 100°C could be caused by the disruption of water molecule interactions, which can be viewed as dissipative structures, and hence far from equilibrium [26, 27]. The ultramolecular dilution behavior after heating at different temperatures has not hitherto been studied in detail in biological systems and particularly in plant models. The only references available are related to the effects of copper sulphate at 15 cH ultramolecular dilution on growth, respiration rate and chlorophyll content of a single-celled green alga, *Chlorella vulgaris* [41]: after heating at 60, 100 and 120°C, the treatment loses its efficacy at all. These results partially disagree with our findings: in fact, in our model the treatment is effective even after heating at 70°C. A detailed study of heating influence on homeopathic treatments would be highly desirable. Indeed, from such study we could derive useful hints, even from a pharmaceutical point of view (for instance for medicine sterilization, storage and transport) and for the purposes of drug compliance, since the potential effectiveness of the homeopathic medicine, even at high environmental temperatures, would make it easy for the patient to preserve.

**Figure 6:**
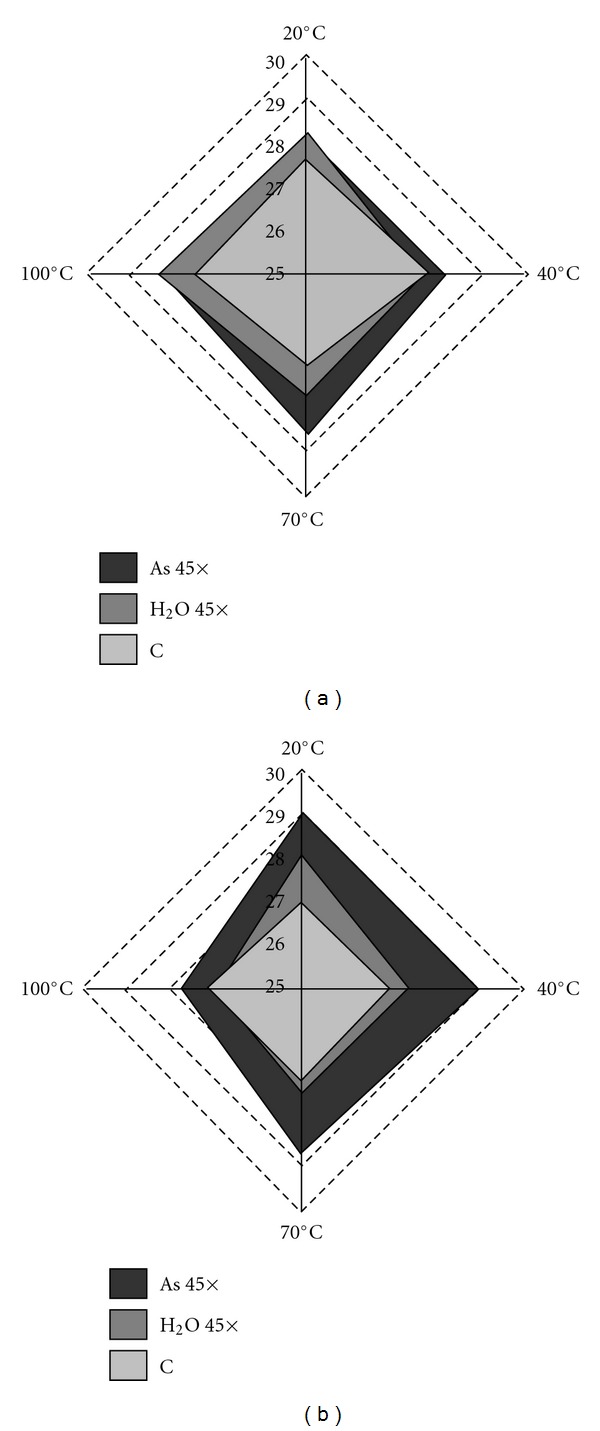
Radar diagram showing the ageing-time effect (a): early trials; (b): late trials on the average number of germinated seeds in control, H_2_O 45× and As 45× treatment groups.

Previous work on physical–chemical properties of ultra-high dilutions as a function of the aging-time parameter have found increases in electrical conductivity and/or heat of mixing [24, 25]. Other conductimetry results have instead found an oscillatory reaction, supporting the idea that ultramolecular dilutions are systems far from equilibrium [26, 27]. In this work we studied the aging-time effect from a biological perspective, a type of approach that has been almost entirely neglected until now. Arsenic at the 45th decimal potency, aged for less than 3 months (early trials), never showed a significant effect on wheat germination (Figure 6(a)), whereas when aged for longer (3–6 months, late trials) the effect became significant at 20, 40 and 70°C (Figure 6(b)). This can be easily observed from the cumulative trend and its linear interpolation, which show strongly differentiated curves for the control (almost constant in time), for H_2_O 45× (slightly decreasing over time) and for As 45× (markedly decreasing over time).

With respect to the effects on variability, the results obtained here for As 45× at 20°C are essentially in line with our previous findings [29], with a remarkably similar percentage reduction in variability versus the control. The reduction was found to affect both components of variability (within and between experiments) and, unlike the effect on mean germination, was observed also in the early trials. This contrasts with a recent work [33] by the Baumgartner research group that examined the effect of gibberellic acid at 17th decimal potency on dwarf pea shoot growth, also in terms of variability, and found it to be slightly reduced only within experiments. However, the experimental context was quite different, with potentized water considered as part of a control group and the gibberellic acid potency below the Avogadro limit, so that it cannot be considered an ultra-high dilution.

The lack of evidence from clinical research on the efficacy of homeopathic remedies as a function of heat or aging raises the question of whether the observations reported here are relevant only to basic research systems or might also have implications for therapeutic use. A similar question is raised by Scherr [34], concerning the continuous relationship between effect and potency level observed in duckweed (*Lemna gibba* L.), a water plant used as a test organism for homeopathic potencies. In our opinion, plant model systems can be of interest not only to answer certain scientific questions about the properties of ultramolecular dilutions but also from a medical point of view, at least as a complement to clinical research.

To summarize, the results of this study show that the efficacy of 45th decimal potency of arsenic trioxide is unaltered at 20 and 40°C and slightly increases at 70°C; if analogous results were detected for other potencies and other substances, it could have potentially useful pharmaceutical and therapeutic implications. Our experimentation on *in vitro* wheat germination model is still in progress: some aspects outlined here, as the strength and shape of the link between temperature and aging, deserve a deeper specifical analysis. Moreover, to give a contribution to understanding the mechanisms of action of homeopathic treatment, we are intended to implement a detailed analysis of some physiological parameters such as enzymatic activities and chlorophyll, soluble sugar and soluble protein contents on wheat seeds (non-stressed, stressed and homeopathically treated after stress).

The results presented here are preliminary to a further research work and do need to be repeated, as independent experiments, a number of times; therefore, definite conclusions on the efficacy of ultramolecular dilutions in specific conditions would be premature. As Bellavite reported [18], finding a rationale and systematic approach to the ultra-high dilution and dynamization phenomena would require a deeper knowledge of the physico-chemical properties of water and the water/alcohol medium.

## Figures and Tables

**Table 1:** Effects of As 45× on germination: comparison between non-stressed and stressed seeds.

Non-stressed seeds						
	1992/93 [19]		1993/94 [21]		1995/96 [21]	
	Germ. rate (%)	Differ. (%)	Germ. rate (%)	Differ. (%)	Germ. rate (%)	Differ. (%)

C	94.6		95.3		93.9	
As 45×	96.8	+2.4**	97.1	+1.9**	96.6	+2.8**

Stressed seeds						

	1992/93 [19]		1993/94 [21]		1995/96 [21]	
	Germ. rate (%)	Differ. (%)	Germ. rate (%)	Differ. (%)	Germ. rate (%)	Differ. (%)

C	n.a.	n.a.	84.8		80.1	
As 45×	n.a.	n.a.	90.2	+ 6.4***	89.8	+12.1***

Germ. = germination; Differ. = difference; n.a. = data not available; [19, 21] = references

**P < .01 (Poisson test); ***P < .001 (Poisson test).

**Table 2:** Negative control experiments: comparison of five experiments at four different temperatures by means of global Poisson test.

	Temperature				*χ* ^2∗^
Experiments	20°C	40°C	70°C	100°C	
1st	37	35	24	26	3.39 n.s.
2nd	24	30	27	28	0.50 n.s.
3rd	28	39	48	35	4.89 n.s.
4th	40	41	37	35	0.41 n.s.
5th	38	28	35	41	2.28 n.s.
*χ* ^2∗∗^	4.51 n.s.	1.61 n.s.	8.99 n.s.	1.66 n.s.	

Each cell value indicates the number of non-germinated seeds out of 198 (six Petri dishes). *χ* ^2∗^ = comparison between temperatures within the same experiment (critical value at *P* = .05: *χ* ^2^ = 7.81). *χ* ^2∗∗^ = comparison between experiments at the same temperature (critical value at *P* = .05: *χ* ^2^ = 9.49).

**Table 3:** Treatments and temperatures: some exploratory statistics.

Class of treatment	Temperature (°C)	N	M(*X*)	SD(*X*)	Me(*X*)	MAD(*X*)
C	20	48	5.708	2.475	5.0	1.792
C	40	48	5.604	2.039	5.5	1.646
C	70	48	5.875	2.288	5.0	1.917
C	100	48	5.646	1.808	6.0	1.479

H_2_O 45×	20	48	4.896	2.321	5.0	1.896
H_2_O 45×	40	48	5.479	1.979	5.0	1.479
H_2_O 45×	70	48	5.396	2.261	5.0	1.938
H_2_O 45×	100	48	5.354	2.665	5.0	1.938

As 45×	20	48	4.500	1.860	4.5	1.458
As 45×	40	48	4.417	2.225	4.5	1.958
As 45×	70	48	4.271	1.668	4.0	1.229
As 45×	100	48	5.042	2.371	5.0	1.958

C = control; *N* = sample size; *X*  = number of non-germinated seeds; M = average; SD = standard deviation; Me = median; MAD = mean absolute deviation from median.

**Table 4:** Global Poisson test for multiple comparisons.

Classes of treatment	Samples compared	Sample size	*χ* ^2^-value	Degrees of freedom	Significance level
C + H_2_O 45× + As 45×	12 (3 × 4 T°)	576	30.317	11	**
C + H_2_O 45×	8 (2 × 4 T°)	384	5.177	7	n.s.
C + As 45×	8 (2 × 4 T°)	384	27.930	7	***
H_2_O 45× + As 45×	8 (2 × 4 T°)	384	15.032	7	*

C = control; T = temperature; n.s. = not significant.

*P < 0.05; **P < .01; ***P < .001.

**Table 5:** Pairwise Poisson test and pairwise Wilcoxon-Mann-Whitney rank sum test at each temperature.

Comparison	Temperature	*N*	Poisson test		Wilcoxon-Mann-Whitney rank sum test	
			Test statistic	*P*-value	Test statistic	*P*-value
C versus H_2_O 45×	20°	48	1.729	.0419*	1.458	0724 n.s.
C versus H_2_O 45×	40°	48	0.260	.3974 n.s.	0.381	.3516 n.s.
C versus H_2_O 45×	70°	48	0.478	.3162 n.s.	1.548	.0609 n.s.
C versus H_2_O 445×	100°	48	0.609	.2712 n.s.	1.103	.1351 n.s.

C versus As 45×	20°	48	2.620	.0044**	2.363	.0091**
C versus As 45×	40°	48	2.599	.0047**	2.301	.0107*
C versus As 45×	70°	48	2.982	.0014**	4.076	< .0001***
C versus As 45×	100°	48	1.280	.1002 n.s.	1.484	.0689 n.s.

H_2_O 45× versus As 45×	20°	48	0.895	.1855 n.s.	0.667	.2524 n.s.
H_2_O 45× versus As 45×	40°	48	2.340	.0096**	1.942	.0261*
H_2_O 45× versus As 45×	70°	48	2.507	.0061**	2.374	.0088**
H_2_O 45× versus As 45×	100°	48	0.671	.2510 n.s.	0.300	.3819 n.s.

C = control; *N* = sample size; n.s. = not significant.

*P < .05; **P < .01; ***P < .001.

**Table 6:** Time effect analysis: pairwise Poisson test for early (1–8) and late (9–16) trials.

Comparison	Temperature	*N*	Early trials (1-8)		Late trials (9-16)	
			Test statistic	*P*-value	Test statistic	*P*-value
C versus H_2_O 45×	20°	24	0.831	.2030 n.s.	1.600	.0548 n.s.
C versus H_2_O 45×	40°	24	−0.251	.4009 n.s.	0.600	.2743 n.s.
C versus H_2_O 45×	70°	24	0.314	.3768 n.s.	−0.369	.3561 n.s.
C versus H_2_O 45×	100°	24	1.224	.1105 n.s.	−0.295	.3840 n.s.

C versus As 45×	20°	24	0.570	.2843 n.s.	3.156	.0008***
C versus As 45×	40°	24	0.514	.3036 n.s.	3.170	.0008***
C versus As 45×	70°	24	1.638	.0507 n.s.	2.571	.0051**
C versus As 45×	100°	24	0.958	.1690 n.s.	0.855	.1963 n.s.

H_2_O 45× versus As 45×	20°	24	−0.260	.3974 n.s.	1.569	.0583 n.s.
H_2_O 45× versus As 45×	40°	24	0.765	.2221 n.s.	2.577	.0050**
H_2_O 445× versus As 45×	70°	24	0.611	.2706 n.s.	1.784	.0372*
H_2_O 445× versus As 45×	100°	24	−0.266	.3951 n.s.	1.150	.1251 n.s.

C = control; *N* = sample size; n.s. = not significant.

*P < .05; **P < .01; ***P < .001.

**Table 7:** Variability of results at 20°C (within and between experiments).

	Class of treatment	*N*_*E*_	SD total	SD within	SD between
All trials	C	8	2.47	2.26	1.01
	H_2_O 45×	8	2.32 (−6.22%)	2.13 (−5.92%)	0.93 (−7.73%)
	As 45×	8	1.86 (−24.85%)	1.62 (−28.19%)	0.91 (−9.95%)

Early trials	C	4	2.63	2.39	1.08
	H_2_O 45×	4	2.36 (−10.28%)	2.11 (−11.79%)	1.05 (−3.24%)
	As 45×	4	1.87 (−28.78%)	1.59 (−33.76%)	0.99 (−8.27%)

Late trials	C	4	2.26	2.12	0.80
	H_2_O 45×	4	2.28 (+0.81%)	2.14 (+1.08%)	0.79 (−1.08%)
	As 45×	4	1.71 (−24.55%)	1.66 (−21.64%)	0.41 (−49.13%)

C = control; *N*_*E*_ = number of experiments; SD = standard deviation.
